# Risk‐minimizing tube current and tube voltage modulation for CT: A simulation study

**DOI:** 10.1002/mp.18047

**Published:** 2025-09-03

**Authors:** Edith Baader, Marc Kachelrieß

**Affiliations:** ^1^ Division of X‐Ray Imaging and CT German Cancer Research Center (DKFZ) Heidelberg Germany; ^2^ Department of Physics and Astronomy Heidelberg University Heidelberg Germany; ^3^ Medical Faculty Heidelberg University Heidelberg Germany

**Keywords:** computed tomography, radiation risk, tube voltage modulation, tube current modulation

## Abstract

**Background:**

The optimal tube voltage in clinical CT depends on the patient's attenuation and the imaging task. Although the patient's attenuation changes with view angle and longitudinal position of the X‐ray tube, the tube voltage remains constant throughout the scan in current clinical practice. In general, the optimum tube voltage increases with patient diameter. For iodine‐enhanced scans, the tube voltage is ideally low to increase contrast. However, 70 kV, the lowest clinically available tube voltage today, can not always be used due to tube current restrictions.

**Purpose:**

To determine the additional relative reduction in effective dose of a tube voltage modulation in addition to a tube current modulation for unenhanced and iodine‐enhanced CT scans.

**Methods:**

For patient models based on CT scans, the effective dose was simulated per projection for different voltages using Monte Carlo simulations. Using these dose data and analytical estimations of noise and iodine contrast, tube voltage and tube current curves were optimized for circular scans. For unenhanced scans, the dose‐weighted noise was minimized, and for iodine‐enhanced scans, the dose‐weighted contrast‐to‐noise ratio (CNRD) was maximized. The effective dose values of the optimized tube voltage and tube current curves (riskTCTVM) were compared at the same noise or same contrast‐to‐noise ratio (CNR) to a pure tube current modulation minimizing the effective dose (riskTCM) and to conventional mAs‐minimizing tube current modulation (mAsTCM).

**Results:**

For unenhanced scans, riskTCTVM reduces the effective dose by less than 1% compared to riskTCM at its optimal tube voltage. For iodine‐enhanced scans, the effective dose benefit increases with the availability of low tube voltages and the eccentricity of the patient's anatomy. For a lowest voltage of 70 kV, we found average effective dose benefits of riskTCTVM to riskTCM of less than 3% for thorax and abdomen, 6% for the pelvis, and 14% for the shoulder. For a lowest voltage of 50 kV, we found average effective dose benefits of 7% for the thorax, 11% for the abdomen, 16% for the pelvis, and 28% for the shoulder. However, the maximum requested tube current was multiple times higher than for mAsTCM at 70 kV. Only for eccentric anatomies in the pelvis and the shoulder, riskTCTVM could lower tube current demands for a lowest available voltage of 70 kV.

**Conclusions:**

For unenhanced scans, tube voltage modulation in addition to a modulated tube current yields a negligible effective dose benefit. However, for iodine‐enhanced circular scans, all studied anatomical regions from shoulder to pelvis would benefit from tube current and tube voltage modulation if X‐ray generators with voltages down to 50 kV were available at sufficient tube power. For a lowest voltage of 70 kV, riskTCTVM can considerably reduce the effective dose for eccentric anatomies in the shoulder and the pelvis.

## 1 INTRODUCTION

To keep the CT radiation risk as low as reasonably achievable (ALARA),^1^ the radiation output of the X‐ray tube should be as dose‐efficient as possible. To achieve this aim, the employed radiation needs to be adapted to the patient's anatomy. Key parameters of so‐called automatic exposure techniques at clinical scanners are the tube current and the tube voltage. The tube current controls the number of emitted photons, which is proportional to the tube current. For a constant tube current, the lowest tube current level achieving the required image quality for diagnosis should be selected. However, more dose‐efficient than a constant tube current is the angular modulation and longitudinal adaptation of the tube current, that is, the tube current is increased for projection angles with high attenuation and decreased for projection angles with low attenuation. Thereby, more homogeneous photon statistics and lower and more homogeneous image noise are achieved compared to a constant tube current at constant mAs‐product; hence, for a certain image quality, the mAs‐product can be lowered resulting in a dose reduction. Depending on the anatomical region, dose reductions of up to 50% can be achieved by using such an mAs‐minimizing tube current modulation (mAsTCM).^2^, ^3^ In clinical CT, mAsTCM techniques have been used now for more than two decades. However, while dose is proportional to the number of photons and therefore to the tube current, simply minimizing the mAs‐product of the scan neglects the actual dose deposition and that different organs have different radiation sensitivities. mAsTCM thus only minimizes the tube output, which typically results in a risk reduction. For a more organ‐specific dose reduction, vendors have developed organ‐specific tube current modulation (TCM) (osTCM) for a few sensitive organs such as the female breast, the thyroid gland, or the eye lens.^4^, ^5^ However, these methods are not patient‐specific, that is, they do not adapt to the patient's anatomy, but decrease the tube current in a predefined angular interval anterior to these organs. To maintain image quality, some osTCM implementations increase the tube current for posterior positions.^4^, ^5^ As several organs contribute to the overall radiation risk, TCM approaches minimizing the effective dose defined as a weighted sum of organ doses^6^ have also been proposed^7^, ^8^ but, to our knowledge, have not been implemented on a scanner yet.

While the tube current controls the number of emitted photons, the tube voltage affects the photons' energy distribution. With increasing tube voltage, the maximum photon energy increases and the energy distribution is shifted towards higher energies. Moreover, the attenuation of the photons depends on the traversed material and the photons' energies. As CT measures the attenuation of X‐rays, there exists an optimal X‐ray energy that depends on the optimization criteria.

For small homogeneous cylinders with diameter D and linear attenuation coefficient μ, one finds that the energy $E_{\text{opt}}$, which fulfills $\mu (E_{\text{opt}})=2/D$ minimizes the number of photons required to achieve a given signal‐to‐noise ratio.^9^ This results in an optimum energy of about 1MeV for a water cylinder with diameter D=30cm.^9^ This energy drops to 70keV if the absorbed dose at the center of the phantom is considered instead of the number of incident photons.^10^ However, while monochromatic X‐ray sources such as synchrotron radiation exist, conventional CT scanners contain X‐ray tubes that emit a spectrum of X‐ray energies. Therefore, later studies focused primarily on the optimal choice of tube voltage.

In one study, the optimal voltage was defined as the one that maximizes dose‐weighted contrast‐to‐noise ratio (CNRD) and was determined for a range of cylindrical water phantoms and quasianthropomorphic phantoms of the thorax and abdomen.^11^ The considered contrasts were calcium, iodine, and an energy‐independent soft‐tissue contrast. The dose in the phantoms was defined as a weighting of peripheral and central dose analogously to CTDI_w_, a commonly used dose measure which is based on measurements in acrylic cylinders. Simulations were performed for 40 kV to 140 kV and measurements for voltages between 60 kV and 140 kV. The authors found that the common settings of 120 kV to 140 kV are suited for soft‐tissue imaging and that voltages below 80 kV are advantageous for calcium (bone) and iodine imaging.

A further study described a workflow for choosing the optimal voltage.^12^ This workflow requires a database generated by preceding phantom measurements or an equivalent mathematical model that can generate the same values. For an image quality parameter, iodine contrast‐to‐noise ratio (CNR) with a noise constraint was proposed. The authors argue that in some iodine‐enhanced exams, there are structures relevant for the diagnosis with a small enhancement that do not benefit from the higher contrast at lower tube voltages. The noise constraint is a parameter in the optimization that reflects how much increase in noise can be tolerated.

Nowadays, modern clinical scanners are equipped with automatic tube voltage selection (ATVS). These ATVS systems retrieve attenuation information from topograms and then select the voltage that results in the lowest dose that stays within the tube current limit for a certain image quality level for a specific imaging task (e.g., soft tissue with or without contrast, CT angiography).^13^

In conclusion, it is recognized that the optimal voltage depends on patient attenuation and the imaging task. However, patient attenuation varies within one scan; while TCM accounts for these changes to use the X‐rays more efficiently, current ATVS systems choose a constant voltage, resulting in a compromise between different view angles. After the introduction of TCM, it was imagined that tube voltage modulation (TVM) could be developed similarly to TCM,^14^ and there are a few proposals of TVM in the literature.

Some TVM techniques were proposed for material testing applications,^15^, ^16^, ^17^ where the differences in attenuation for different view angles can be enormous. Also, for cone‐beam C‐arm systems, a tube voltage modulation can be beneficial.^18^, ^19^, ^20^ In the field of clinical CT, a variation of the tube voltage throughout the scan was also proposed to perform material decomposition with a conventional single‐energy CT system.^21^, ^22^, ^23^ For photon‐counting CT systems, TVM has also been studied to reduce noise in material decomposition tasks.^24^

Regarding a dose reduction in clinical CT, one study investigated the potential reduction in breast dose by using different voltages for anterior and posterior views.^25^ However, this study lacked an investigation of the image quality, and therefore, the potential dose reduction at constant image quality for this strategy remains unknown. With this study, we want to investigate the potential of a tube voltage modulation in addition to TCM to reduce the effective dose relative to TCM for unenhanced scans at constant noise and for iodine‐enhanced scans at constant CNR.

## 2 MATERIALS AND METHODS

### 2.1 General concept

For the optimization of our combined tube current and tube voltage modulation, we consider circular scans with a tube current value I(α) and a tube voltage value U(α) for every projection angle α. For a given modulation, the effective dose for these parameter settings is calculated using a patient and slice‐specific look‐up table. Furthermore, the noise inside the patient's body and the contrast of a simulated inserted iodine disk in the center of the considered slice are estimated. Subsequently, the resulting CNRD is calculated. To maximize the CNRD, the tube current and tube voltage curves are optimized using the L‐BFGS‐B algorithm.^26^, ^27^ For scans without contrast material or for TCMs at constant voltage, we minimize the dose‐weighted noise, that is, the denominator of the CNRD.

The following sections are organized as follows: Section 2.2 describes the used patient models, Section 2.3 describes the determination of the effective dose per scan using Monte Carlo dose simulations, Section 2.4 describes the noise estimation, and Section 2.5 describes the contrast estimation. The optimization, baseline TCMs, and the calculation of relative effective doses at constant noise or constant CNR are described in Section 2.6.

### 2.2 Patient models

**FIGURE 1:**
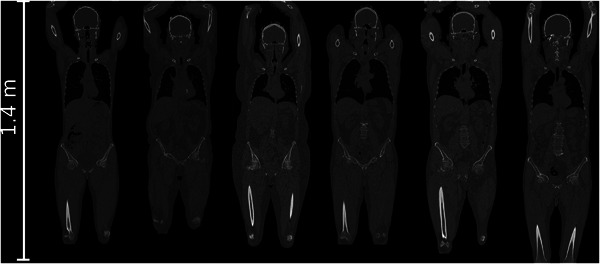
Coronal views of the considered three female (left) and three male (right) patients from the Visceral dataset.^28^

Our patient models are based on CT datasets of three female and three male patients from the Visual Concept Extraction Challenge in Radiology (Visceral)^28^ (see Figure 1). The CT scans were acquired unenhanced and have a field of view from the head to the knee of the patients.^28^ Organ segmentations that were created for the study of a risk‐minimizing tube current modulation (riskTCM) as described in reference 7 were used for the calculation of organ doses.

### 2.3 Dose model

#### Per‐view dose simulations

For the dose simulations, we assign every voxel of the patients' CT volume to one of five materials (air, lung tissue, fat, water, bone) using a threshold‐based segmentation and calculate the corresponding density from the CT value. With our in‐house Monte Carlo dose simulation software, we create dose distributions for tube positions

s(α,z)=+RFsinα−RFcosαzwithRF=595mm

on a circular trajectory, that is, at a fixed longitudinal table position z and projection angles α covering $360^{\circ }$ with an angular increment of $10^{\circ }$. In the following, we drop the dependency on z for better readability. Simulations were performed for longitudinal positions z from the shoulder to the pelvis of the patients with an increment of about 38mm. We consider the scanner geometry of a Somatom Definition Flash scanner with a collimation of 38.4mm, a source‐to‐isocenter distance of $R_{F}=595\;\mathrm{mm}$, a source‐to‐detector distance of 1085.6mm, and a field of measurement of 500mm. Furthermore, the photons are attenuated by a bowtie filter. We simulate the dose distributions for photon energy intervals of 5keV from 20 keV to 150keV. To calculate the per‐view effective dose for arbitrary tube voltages, we use Tucker spectra^29^ with 6mm aluminum prefiltration. We then weigh the dose values for the different simulated photon energies according to these Tucker spectra.

#### Calculation of per‐view effective dose

From the per‐view dose distributions D(α,r), we obtain organ doses $D_{T}\left(\alpha \right)$ by calculating the mean organ dose, that is, the total deposited energy in that organ divided by the organ mass, which is given by the integral of the density ρ(r) over the organ volume:^6^

$$D_{T}\left(\alpha \right)=\frac{\int_{T}D\left(\alpha ,r\right)\rho \left(r\right)dV}{\int_{T}\rho \left(r\right)dV}.$$

Building a weighted sum of these organ doses with organ weighting factors $w_{T}$, we calculate per‐view effective doses by

$$D_{\text{eff}}\left(\alpha \right)=\sum_{T}w_{T}D_{T}\left(\alpha \right).$$

**FIGURE 2:**
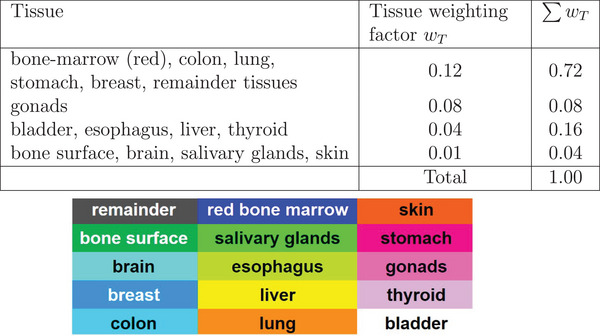
Tissue weighting factors as recommended by the ICRP^6^ and color scheme used in this study to label the different organs.

We consider all 14 organs with a weighting factor equal to or greater than 0.01 in the recommendations of the International Commission on Radiological Protection (ICRP)^6^ (compare table in Figure 2). All other tissues were treated as one single organ and assigned the weighting factor for the remainder tissues.

Finally, we weigh the obtained per‐view effective doses $D_{\text{eff}}\left(\alpha \right)$ for the photon energy intervals according to their intensity in the generated Tucker spectra to obtain per‐view effective doses $D_{\text{eff}}\left(\alpha ,U\right)$ for different voltages U. The created Tucker spectra model the photon output per electron considering that different voltages result in different numbers of photons per electron.

#### Calculation of effective dose per scan

To obtain the overall effective dose for a circular scan, we build the sum over the per‐view effective doses multiplied by the respective tube current I(α):

$$D_{\text{eff}}\left(I,U\right)=\sum_{\alpha }I\left(\alpha \right)\cdot D_{\text{eff}}\left(\alpha ,U\left(\alpha \right)\right).$$

### 2.4 Noise estimation

To estimate the image noise, we need to propagate the expected noise in the raw data domain to the image domain. For every intersection through the patient, we consider a water‐equivalent path‐length $l_{W}$ for water of density $\rho_{W}=1\;g\;{\mathrm{cm}}^{-3}$ with mass attenuation coefficient $\psi_{W}\left(E\right)$ and an intersection through iodine with density $\rho_{I}=4.93\;\mathrm{mg}\;{\mathrm{cm}}^{-3}$ with mass attenuation $\psi_{I}\left(E\right)$. This leads to a polychromatic attenuation value q for the projection angle α and the angle β within the fan of

(1) $$\begin{array}{ccc} q(\alpha ,\beta ) & = & -\ln\int \mathrm{dE}\;w(E,U,\beta )\\ & & \cdot \;e^{-l_{W}\left(\alpha ,\beta \right)\psi_{W}\left(E\right)-l_{I}\left(\alpha ,\beta \right)\psi_{I}\left(E\right)}. \end{array}$$

Here, E denotes the photon energy and w(E,U,β) the normalized detected spectrum. We model the detected spectrum $n_{0}\left(E,U,\beta \right)$ as follows

n0(E,U,β)=nT(E,U)︸Tucker spectrum·nTe−μB(E)·dB(β)︸Bowtie·nTE·(1−e−μD(E)dD)︸Detector.

The first term n(E,U) denotes the voltage‐dependent Tucker spectrum^29^ with 6mm aluminum prefiltration. The second term models the attenuation by the bowtie filter, and the third term the detection of an energy‐integrating detector for which we consider a $d_{D}=1.4\;\mathrm{mm}$ thick gadolinium oxysulfide scintillator layer with a density of $7.32\;g\;{\mathrm{cm}}^{-3}$. We denote the integral of the detected spectrum over the energy with $N_{0}\left(U,\beta \right)$, that is, $N_{0}\left(U,\beta \right)=\int dEn_{0}\left(E,U,\beta \right)$. Consequently, the normalized detected spectrum is given by $w\left(E,U,\beta \right)=n_{0}\left(E,U,\beta \right)/N_{0}\left(U,\beta \right)$.

We obtain the water‐equivalent intersection lengths by forward projection of the density of the patient CT volume and dividing by $\rho_{W}=1\;g\;{\mathrm{cm}}^{-3}$. As in reference 7, we perform the forward projection for different detector rows and perform an average over the detector width, that is

$$l_{W}\left(\alpha ,\beta \right)=\frac{1}{B}\int_{-B/2}^{+B/2}db\;l_{W}\left(\alpha ,\beta ,b\right)$$

where B denotes the width of the detector in the isocenter, that is, the collimation.

The detected signal S at the detector is described by $S=I\cdot N_{0}\cdot e^{-q}$. Following the derivation of image noise of reference 7, we assume this signal to be Poisson‐distributed, that is, VarS=S. By error propagation and assuming no uncertainty on I and $N_{0}$, this yields a variance of the polychromatic attenuation of

$$\text{Var}\;q=\frac{e^{q}}{I\cdot N_{0}}\;\text{for}\;q=\ln\left(S\right)-\ln\left(I\cdot N_{0}\right).$$

For image reconstruction, the measured polychromatic attenuation values are water‐precorrected. Water precorrection relates the polychromatic attenuation values to single intersection lengths with water p:

(2) $$q=Q\left(p\right)=-\ln\;\;\int \;\;dE\;w\left(E,U,\beta \right)e^{-p\psi_{W}\left(E\right)}.$$

Equation (2) is a bijective relation between p and q, and we denote the water precorrection function with p=P(q,w). We perform the water precorrection p=P(q,w) using tabulated values that we create for every spectrum w:=w(E,U,β), that is, for every voltage U and every angle β within the fan.

Propagating the noise of q to p yields

(3) $$\text{Var}p\propto \frac{e(p,w)}{I\cdot N_{0}}\;\text{with}\;e\left(p,w\right)={\left(P^{\prime }\left(Q\left(p\right),w\right)\right)}^{2}e^{Q(p)}.$$

This relation was similarly derived in reference 7. Following reference 7, we assume that complementary rays are weighted in a statistically optimal way, that is, inversely to their variance. Thus, the variance of the projection value for two complementary rays at source positions $\alpha_{C}$ and $\alpha_{D}$ is

$$\begin{array}{cc} V_{\text{opt}} & =\frac{1}{1/V_{D}+1/V_{C}}\\ & ={\left(\frac{I_{D}\cdot N_{0}\left(U_{D},+\beta \right)}{e(p\left(\alpha_{D},+\beta \right),w\left(U_{D},+\beta \right))}+\frac{I_{C}\cdot N_{0}\left(U_{C},-\beta \right)}{e(p\left(\alpha_{C},-\beta \right),w\left(U_{C},-\beta \right))}\right)}^{-1} \end{array}$$

The statistically optimal weighting of complementary rays allows the optimization to turn off the tube current for projection angles with a high effective dose.

For the optimization, we consider the image noise throughout the whole patient body. Similar to reference 7, we compute the overall noise as the weighted sum of noise values for different positions across the patient's body:

(4) $$\begin{array}{ccc} & & N(I,U)=\int dxdy\;w(r)\\ & & \cdot \;\sqrt{\int d\vartheta {\left(\frac{I_{D}\cdot N_{0}\left(U_{D},+\beta \right)}{e(p\left(\alpha_{D},+\beta \right),w\left(U_{D},+\beta \right))}+\frac{I_{C}\cdot N_{0}\left(U_{C},-\beta \right)}{e(p\left(\alpha_{C},-\beta \right),w\left(U_{C},-\beta \right))}\right)}^{-1}}. \end{array}$$

Here, w(r) denotes the noise weight for different locations r in the image. In this study, we set this weight to one inside the patient and zero outside. However, w(r) could also be used to enhance the image quality for specific anatomical regions for a specific diagnostic task. The angle ϑ denotes here the angle of a ray going through the point r=(x,y) in parallel beam geometry. The distance of that ray to the isocenter is then given by ξ=xcosϑ+ysinϑ. The corresponding tube angle α and fan angle β can be retrieved using the equations ϑ=α+β and $\xi =-R_{F}\sin\beta$.

### 2.5 Contrast estimation

To investigate the benefit of a tube voltage modulation for contrast‐enhanced scans, we artificially insert an iodine disk of 2cm diameter and density $\rho_{I}=4.93\;\mathrm{mg}\;{\mathrm{cm}}^{-3}$, that is, iodine diluted by a factor of 1000, into the CT images in the isocenter.

The water precorrection assumes that the whole patient consists of water. Hence, the contrast in the reconstructed image corresponds to the density that water would require to exhibit the same attenuation as the contrast material. We can therefore add the following third expression of the polychromatic attenuation value q to Equations (1) and (2):

$$\begin{array}{cc} q & =-\ln\;\;\int \;\;dE\;w\left(E,U,\beta \right)e^{-l_{W}\psi_{W}\left(E\right)-l_{I}\psi_{I}\left(E\right)}\\ & =-\ln\;\;\int \;\;dE\;w\left(E,U,\beta \right)e^{-p\psi_{W}\left(E\right)}\\ & =-\ln\;\;\int \;\;dE\;w\left(E,U,\beta \right)e^{-\left(l_{W}+l_{I}C_{I}\right)\psi_{W}\left(E\right)}. \end{array}$$

We can hence calculate the contrast by solving the equation

$$p=l_{W}+l_{I}C_{I}$$

which gives us

(5) $$C_{I}=\frac{p-l_{W}}{l_{I}}.$$

The contrast in the isocenter is then given by the sum of the contrast values (defined by Equation 5) for the central rays with complementary rays weighted by their variance.

### 2.6 Optimization

#### Unenhanced scans

For unenhanced scans, we minimize the dose‐weighted noise which we define by

(6) $$N_{D}=\sqrt{D/D_{\text{ref}}}\cdot N\;\text{with}\;D≔D_{\text{eff}}.$$

This definition is invariant against scalings of the tube current as the dose is proportional to the current and the noise antiproportional to the square root of the current. Thus, regarding the tube current, only the relative course is relevant for the optimization. For the voltage, we consider a voltage range of 70 kV to 150 kV. After the optimization, the tube current can be scaled to achieve a certain noise. Solving Equation (6) for the dose, the dose for any desired noise is given by

$$D={\left(\frac{N_{D}}{N}\right)}^{2}\cdot D_{\text{ref}}$$

and we can therefore calculate the relative dose to a reference modulation with $N_{D,\text{ref}}$ by

$$\text{Relative dose}=\frac{N_{D}^{2}}{N_{D,\text{ref}}^{2}}.$$

We then define the dose reduction compared to the reference modulation as

Dose reduction=1−Relative dose.

#### Iodine‐enhanced scans

For iodine‐enhanced scans, we maximize the CNRD defined by

(7) $$\text{CNRD}=\frac{C}{\sqrt{D/D_{\text{ref}}}\cdot N}.$$

Again, this definition is invariant to scalings of the tube current. For the tube voltage, we set an upper bound of 150 kV. For the lowest allowed voltage, we consider four different scenarios, namely 40 kV, 50 kV, 60 kV, and 70 kV. For optimization, we formulate the optimization of the CNRD as a minimization of the negative CNRD.

The relative dose at constant CNR is given for enhanced scans by

$$\text{Relative dose}=\frac{{\text{CNRD}}_{\text{ref}}^{2}}{{\text{CNRD}}^{2}}$$

#### Discretization

We optimize the tube current and the tube voltage for circular scans with a spacing of $1^{\circ }$. For the calculation of noise and contrast, we create sinograms with an angular spacing of $1^{\circ }$ for the tube angle α and $0.05^{\circ }$ for the angle within the fan β. To obtain dose values at a pitch of $1^{\circ }$, we interpolate the simulated dose values. For the calculation of the overall noise (compare Equation 4), we evaluate the noise at points (x,y) on a grid with a spacing of approximately 7mm. The integral over the angle ϑ (compare Equation 4) is performed in steps of $0.5^{\circ }$.

#### Optimization algorithm

For the optimization, we use the implementation of the L‐BFGS‐B^26^, ^27^ algorithm provided within the optimization framework of SciPy.^30^ The L‐BFGS‐B algorithm builds upon the Broyden–Fletcher–Goldfarb–Shanno (BFGS) algorithm and allows for bounds on the input variables and operates with limited memory.^26^ Furthermore, the L‐BFGS‐B algorithm is a quasi‐Newton method that uses an approximate of the Hessian matrix.^26^ We therefore compute besides the objective function values, that is, the dose‐weighted noise or the CNRD, only the gradients with respect to every tube current and every tube voltage value.

#### Baseline tube current modulations

We consider two TCM methods as a baseline to riskTCTVM, namely riskTCM and mAsTCM. For riskTCM, we minimize the dose‐weighted noise as defined by Equation (6). We perform this optimization for voltages between 40 kV and 150 kV in steps of 5 kV. For mAsTCM, we calculate the tube current values analytically for the same voltages as described in the following. For minimizing the image noise in the center at constant mAs‐product, the optimal tube current is proportional to the square root of the attenuation.^2^ Considering polychromatic attenuation undergoing a water precorrection, the following expression for the optimal tube current in the central ray approximation was derived in reference 7:

$$I_{\text{mAsTCM}}\left(\alpha \right)\propto \sqrt{e(p(\alpha ),w)}$$

with the definition of e(p) as defined by Equation (3). As in reference 7, we use the 90th percentile of p(α,β) as a surrogate for p(α) in the central ray approximation, and we use the detected spectrum w for the central ray with β=0.

## 3 RESULTS

### 3.1 Dose‐weighted noise minimization

**FIGURE 3:**
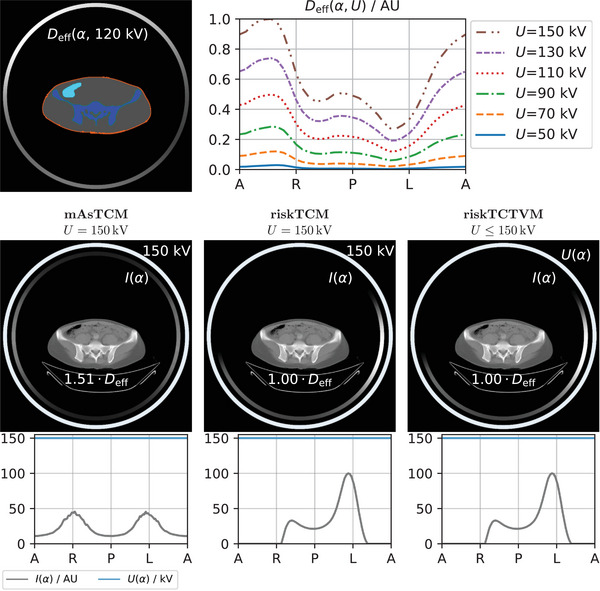
Dose‐weighted noise optimization for a slice in the pelvis of a female patient. The top row shows the organ segmentation and per‐view effective dose as a function of tube angle α and tube voltage U. The labeling of the x‐axis corresponds to the tube positions anterior (A), right (R), posterior (P), left (L), and anterior (A) from the patient's perspective. Below, the resulting modulations for mAsTCM, riskTCM, and riskTCTVM are shown. The effective dose values are given for a constant noise level and relative to riskTCM at the best voltage for this anatomy which is U=150kV.

Figure 3 shows an example of the dose‐weighted noise minimization for a slice in the pelvis. mAsTCM considers only the attenuation of the patient and increases the tube current for lateral views, which have a higher attenuation than anterior‐posterior views due to the eccentric body shape. riskTCM instead also considers the high effective dose for views from the right and anterior to the patient due to the colon; therefore, riskTCM turns the tube current off for these positions and increases the tube current on the left side of the patient. For mAsTCM and riskTCM, the optimal voltage is 150 kV; this means that the noise per effective dose is lowest for 150 kV. The effective dose per unit tube current, however, is highest for 150 kV (see Figure 3). The different tube current distribution of riskTCM results in a decrease in the effective dose of approximately 34% compared to mAsTCM. riskTCTVM also selects 150 kV for the full rotation resulting in the same tube current curve and the same effective dose as riskTCM. For all patients and anatomical regions that we studied, we observed at most slight deviations in the tube voltage to voltages below 150 kV; however, also in the cases with slight deviations in the voltage, the effective dose benefit compared to riskTCM was well below 1%.

### 3.2 CNRD optimization

#### 3.2.1 Example modulations

**FIGURE 4:**
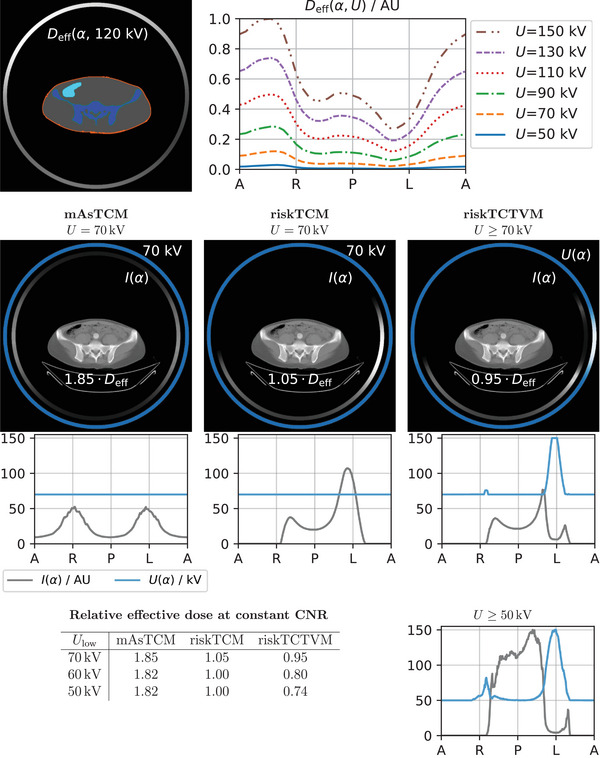
CNRD optimization for a slice in the pelvis of a female patient. The effective dose values are given at constant CNR and relative to riskTCM at its best voltage which is 60 kV for this anatomy.

Figure 4 shows the CNRD optimization for the same slice as in Figure 3. The effective dose values are given for a constant CNR and relative to riskTCM for the voltage with the highest CNRD. For the CNRD optimization, lower voltages are beneficial compared to pure noise optimization, as lower voltages increase the iodine contrast. Here, the effective dose benefit of riskTCTVM compared to riskTCM highly depends on the allowed voltage range in the optimization. For a lowest allowed voltage of $U_{\text{low}}=70\;\mathrm{kV}$, riskTCTVM decreases the effective dose at constant CNR about 10% compared to riskTCM at constant 70 kV. For a lowest allowed voltage of 60 kV, this benefit in effective dose increases to 20% compared to riskTCM at 60 kV. which is the optimal voltage for riskTCM; a decrease of the lowest voltage to 50 kV thus does not offer further decrease of effective dose for riskTCM. riskTCTVM instead lowers the voltage for some angular range to 50 kV which increases the effective dose benefit to 26% compared to riskTCM at 60 kV. For a lowest voltage of 40 kV, we observed no or only slight further effective dose reductions of up to 3% for all anatomies we studied.

**FIGURE 5:**
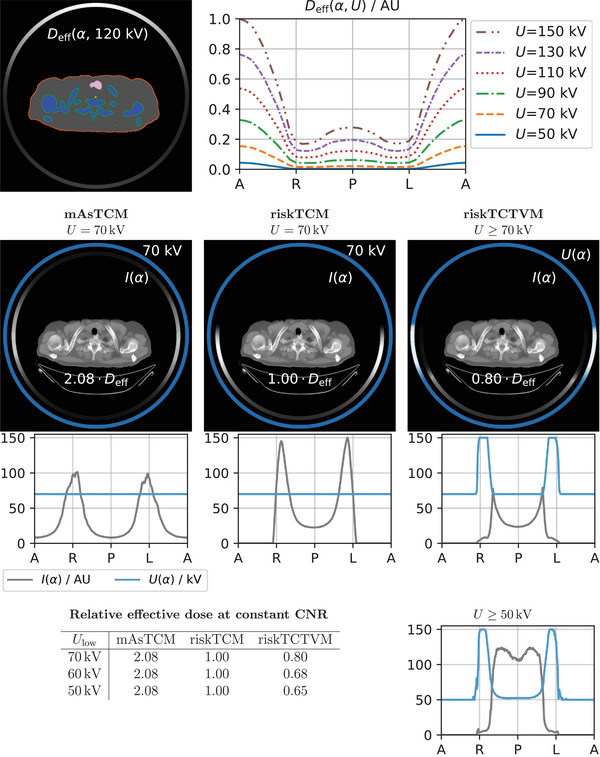
CNRD optimization for a slice in the shoulder of a male patient. The effective dose values are given at constant CNR and relative to riskTCM at its best voltage which is 70 kV for this anatomy.

Figure 5 shows the CNRD optimization for a slice in the shoulder for which we found the highest relative effective dose reduction of riskTCTVM compared to riskTCM. For this anatomy, riskTCTVM decreases the effective dose by about 35% compared to riskTCM for the lowest allowed voltage of $U_{\text{low}}=50\;\mathrm{kV}$. For $U_{\text{low}}=70\;\mathrm{kV}$, the decrease in effective dose is 20%. Remarkable for this voltage range is also the decrease in the maximum requested tube current. Although riskTCTVM irradiates the patient from a smaller angular range than mAsTCM, the maximum tube current is lower due to the partial increase of the tube voltage to 150 kV.

**FIGURE 6:**
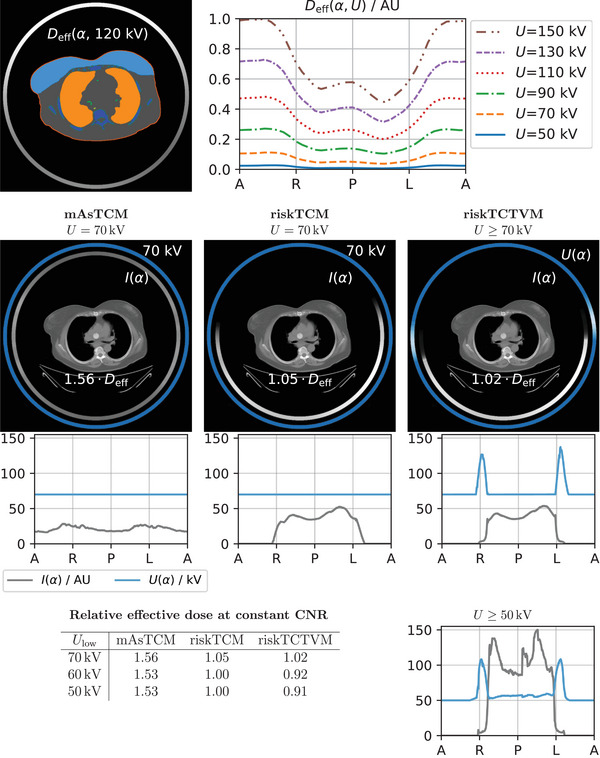
CNRD optimization for a slice in the thorax of a female patient. The effective dose values are given at constant CNR and relative to riskTCM at its best voltage which is 60 kV for this anatomy.

**FIGURE 7:**
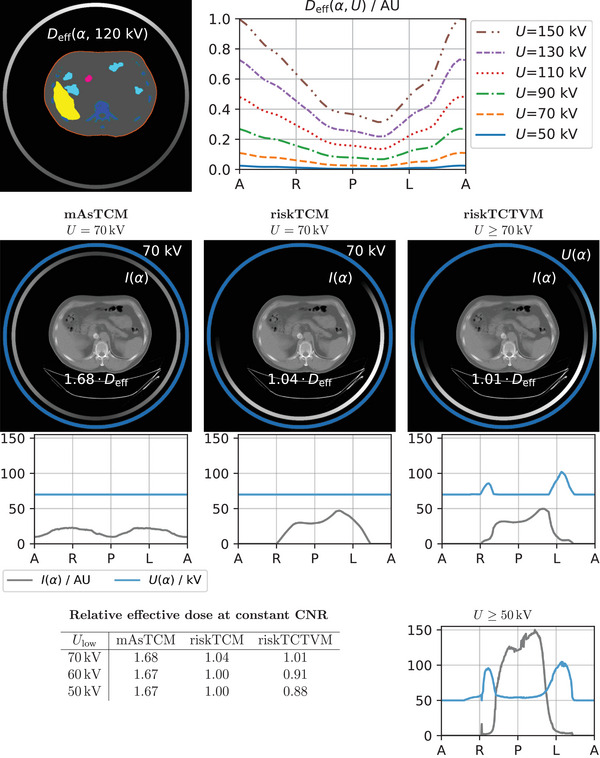
CNRD optimization for a slice in the abdomen of a male patient. The effective dose values are given at constant CNR and relative to riskTCM at its best voltage which is 60 kV for this anatomy.

**FIGURE 8:**
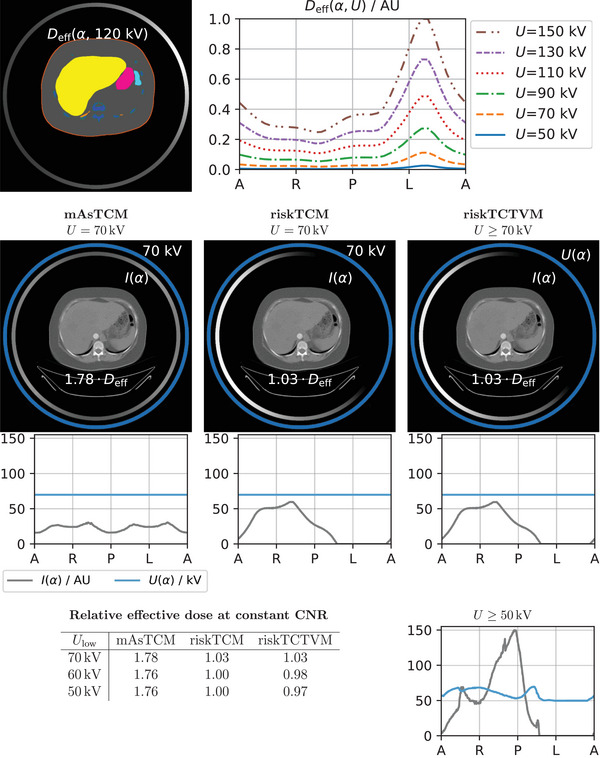
CNRD optimization for a slice in the abdomen of a female patient. The effective dose values are given at constant CNR and relative to riskTCM at its best voltage, which is 60 kV for this anatomy.

Figures 6 and 7 show example slices in the thorax and abdomen for which the maximum effective dose benefits of riskTCTVM compared to riskTCM are 9% and 12% and thus lower than for the examples of the abdomen and the shoulder. Furthermore, Figure 8 shows a case in the abdomen for which riskTCTVM exhibits no effective dose benefit for a lowest voltage of 70 kV and an effective dose benefit of only 3% for a lowest allowed voltage of 50 kV.

#### 3.2.2 Relative effective dose

**FIGURE 9:**
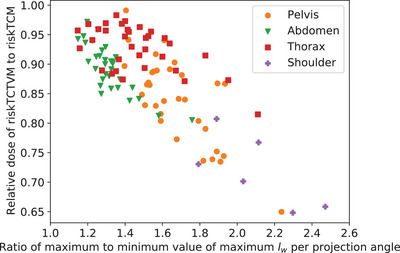
Effective dose of riskTCTVM relative to the effective dose of riskTCM for $U_{\text{low}}=50\;\mathrm{kV}$ for different body regions at constant iodine CNR. The *x*‐axis shows the ratio of the maximum to the minimum value of the maximum water intersection length $l_{W}$ per projection angle.

Figure 9 shows the effective dose values of riskTCTVM relative to the effective dose of riskTCM for a lowest voltage of 50kV for all slices of all patients that we studied. The *x*‐axis shows the ratio of the maximum to the minimum value of the maximum water intersection length $l_{W}$ per projection angle. As especially visible in Figures 4 and 5, riskTCTVM tends to increase the voltage for angles for which mAsTCM increases the tube current. As Figure 9 reveals, the relative effective dose benefit of riskTCTVM compared to riskTCM correlates with the ratio of the maximum to the minimum value of the maximum water intersection length $l_{W}$ per projection angle; this correlation explains in part the differences between the mean values for the different body regions (Table 1). For example, the relative effective dose benefits for the shoulder and the pelvis are on average higher than for the thorax and the abdomen which are less eccentric body regions and thus exhibit lower ratios of maximum to minimum water intersection length. The highest average decrease in effective dose of riskTCTVM relative to riskTCM we observe for the shoulder; here, the average effective dose decrease is 14% for a lowest voltage of 70 kV and 28% for a lowest voltage of 50kV ranging from 19% to 35%. For one patient, we also found a dose decrease of 35% for one slice in the pelvis but the average value for the pelvis is 16% and thus lower than for the shoulder presumably due to the wider spread of the eccentricity of the body shape in the pelvis compared to the shoulder.

**TABLE 1:** Effective dose values of the investigated modulation techniques relative to those of riskTCM for riskTCM's optimal tube voltage (i.e., the tube voltage for which riskTCM achieves the lowest dose).

	$U_{\text{low}}$	mAsTCM	riskTCM	riskTCTVM
Shoulder	70 kV	1.84 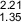	1.01 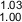	0.87 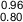
	60kV	1.84 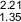	1.00 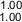	0.75 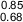
	50kV	1.84 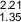	1.00 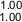	0.72 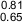
Thorax	70 kV	1.36 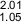	1.04 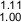	1.02 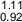
	60kV	1.33 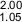	1.00 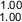	0.94 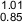
	50kV	1.33 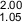	1.00 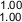	0.93 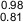
Abdomen	70 kV	1.73 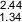	1.06 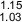	1.05 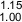
	60kV	1.69 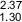	1.00 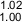	0.94 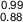
	50kV	1.69 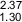	1.00 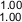	0.89 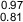
Pelvis	70 kV	1.52 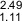	1.03 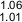	0.97 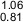
	60kV	1.50 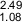	1.00 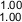	0.87 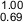
	50kV	1.50 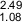	1.00 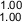	0.84 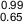

*Note*: All values are mean values over all patients and slices given for a constant iodine CNR. The values in sub‐ and superscript denote the minimum and maximum values. $U_{\text{low}}$ refers to the lowest tube voltage we allow in our simulation.

#### 3.2.3 Maximum requested tube current

**FIGURE 10:**
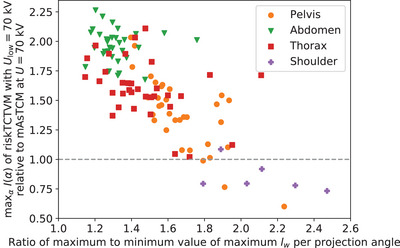
Maximum tube current requested by riskTCTVM for $U_{\text{low}}=70\;\mathrm{kV}$ relative to mAsTCM at U=70kV for different body regions at constant iodine CNR. The *x*‐axis shows the ratio of the maximum to the minimum value of the maximum water intersection length $l_{W}$ per projection angle.

In clinical practice, the optimal voltage can not always be selected due to tube current constraints. In these cases, a higher tube voltage is chosen. Therefore, we also compared the maximum tube current requested by riskTCTVM to the maximum tube current of mAsTCM at 70 kV, the lowest available tube voltage in current clinical practice. Figure 10 shows the maximum tube current requested by riskTCTVM for a lowest voltage of 70 kV relative to the maximum tube current of mAsTCM at 70 kV. For anatomies in the abdomen and thorax, riskTCTVM for $U_{\text{low}}=70\;\mathrm{kV}$ requests maximum tube current values of up to 226% and 211% of the maximum tube current of mAsTCM for U=70kV. For eccentric anatomies in the shoulder and the pelvis, the maximum tube current requested by riskTCTVM is instead lower for riskTCTVM. Table 2 summarizes the results for the maximum requested tube current for the different body regions and different minimum voltages. Only for the shoulder, the maximum tube current requested for riskTCTVM for $U_{\text{low}}=70\;\mathrm{kV}$. is on average, 15% lower than for mAsTCM at 70 kV. For all other body regions and voltages, the maximum requested tube current, is on average, higher.

**TABLE 2:** Maximum tube current values of the investigated modulation techniques relative to those of mAsTCM at 70 kV.

	$U_{\text{low}}$	mAsTCM	riskTCM	riskTCTVM
Shoulder	70 kV	0.92 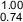	1.50 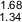	0.85 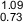
	60kV	0.92 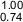	2.08 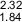	1.15 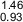
	50kV	0.92 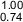	2.08 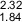	1.95 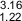
Thorax	70 kV	1.00 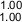	1.63 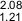	1.60 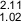
	60kV	1.38 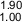	2.68 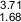	2.90 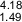
	50kV	1.38 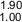	2.68 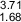	4.91 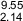
Abdomen	70 kV	1.00 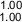	1.91 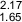	1.97 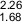
	60kV	1.44 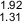	3.65 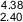	3.55 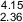
	50kV	1.44 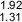	3.86 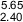	5.82 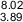
Pelvis	70 kV	1.00 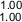	1.61 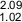	1.39 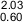
	60kV	1.32 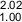	2.53 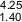	2.01 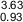
	50kV	1.32 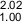	2.53 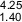	2.59 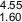

*Note*: All values are mean values over all patients given for a constant iodine CNR. The values in sub‐ and superscript denote the minimum and maximum values. $U_{\text{low}}$ refers to the lowest tube voltage we allow in our simulation. For mAsTCM, average values below 1 occur for the shoulder because the optimal voltage for mAsTCM is for some anatomies in the shoulder 75kV.

## 4 DISCUSSION

For the dose‐weighted noise optimization, we found negligible effective dose benefits of less than 1% of riskTCTVM compared to riskTCM at constant noise. For a dose‐weighted iodine CNR maximization, the effective dose benefit of riskTCTVM highly depends on the lowest available voltage. For a lowest allowed voltage of 50kV, average reductions in effective dose by riskTCTVM compared to riskTCM are between 7% for the thorax and 28% for the shoulder. However, the maximum requested tube current is in most cases several times higher than for mAsTCM at 70 kV. For $U_{\text{low}}=70\;\mathrm{kV}$, the maximum requested tube current of riskTCTVM is instead on average 15% lower than for mAsTCM at 70 kV. Furthermore, the decrease in effective dose compared to mAsTCM is about 50%. While riskTCM at 70 kV also greatly reduces the effective dose in the shoulder compared to mAsTCM, the maximum requested tube current is on average 50% higher than for mAsTCM, which makes the application of riskTCM at 70 kV more demanding.

### 4.1 Study limitations

The study included several simplifications. For the CNRD optimization, we artificially introduced a cylinder filled with iodine of constant density in the isocenter. This allowed us to study general effects on the riskTCTVM optimization for the choice of voltages in the presence of iodine contrast. Furthermore, the iodine cylinder could serve as a simple model for a blood vessel filled with iodine. Future studies including variations of the inserted contrast density, size, and location could give insight into the relevance of these parameters for the optimized curves and the dose benefits.

Also, the patient cohort should be considered when interpreting the results. The study included patients of different sex, for example, three female and three male patients, and of different patient diameters (compare Figure 1). The study thus showed the benefit of riskTCTVM for a range of patients differing in relevant characteristics for risk reduction. For statements about the general population or a specific patient population, however, a higher number of patients with a representative composition, as in the population of interest, should be studied. Nevertheless, the observed correlation of the effective dose benefits with the eccentricity of the anatomy could indicate the expected dose benefits for other patients. Especially for very eccentric anatomies, such as the shoulder region, riskTCTVM showed promising decreases in effective dose and maximum tube current for all patients.

Moreover, this work is a simulation study. Ideally, scans with the optimized tube voltage modulations would be acquired on a clinical scanner to validate the simulation results. However, the application of tube voltage modulation on a clinical scanner requires several prerequisites as discussed in the following.

### 4.2 Prerequisites for the application on a clinical scanner

In our simulation study, we assume that choices of the tube voltages in 1kV steps are possible. While the voltage remains constant throughout the CT scan for most systems, CT scanners with fast kV‐switching are available. These systems switch between two different voltages in less than a millisecond for the acquisition in dual‐energy CT.^31^ The availability of such fast kV‐switching techniques therefore seems promising for the realization of continuous voltage changes at a slower rate as proposed by the riskTCTVM optimization. However, a calibration for every voltage has to be available in order to perform water precorrection. Here, the application of proposed calibration techniques for systems with tube voltage modulation^18^, ^19^ could be considered.

Another crucial factor for our contrast optimization is the availability of low tube voltages. Currently, the lowest voltage on clinical scanners is typically 70 kV. However, a scanner with 60kV was provided for the study in reference 11. In practice, however, tube current limits can prohibit the use of low voltages, such as 70 kV. even if the tube voltage is available in principle. This is because lower tube voltages require a higher tube current to achieve the same image noise and this increase in tube current can be above the tube current limit of the X‐ray tube, depending on the anatomy and the desired noise level. In our simulation study, the maximum requested tube current of riskTCTVM was higher than the one for mAsTCM at 70 kV except for eccentric slices in the pelvis and most slices in the shoulder for $U_{\text{low}}=70\;\mathrm{kV}$. For these cases, the application of riskTCTVM seems feasible as scans at 70 kV can be performed in the shoulder with powerful X‐ray tubes which allow tube currents of up to 1300mA.^32^, ^33^, ^34^ For other scan scenarios, the inclusion of a tube current limit in the optimization would be beneficial to study the possible effective dose reductions given a maximum tube current limit.

For the estimation of the effective dose or other risk measures based on organ doses, several prerequisites are needed. The first prerequisite is a 3D reconstruction of the patient. As discussed in reference 7, this reconstruction could be obtained by an estimation from 2D topograms.^35^, ^36^, ^37^, ^38^ Another option could be a low‐dose 3D scout scan.^39^, ^40^ Second, an organ segmentation is needed for the calculation of organ doses which could be retrieved with deep learning models.^41^ Finally, a fast dose estimation is necessary to estimate the dose deposition beforehand. This could also be based on deep learning models as the deep dose estimation^42^ employed for the study of riskTCM^7^ that was trained to reproduce Monte Carlo simulations. Another option could be fast analytical dose estimations combining first‐order estimates with convolution kernels to approximate higher‐order interactions.^43^

## 5 CONCLUSION

For unenhanced scans, riskTCTVM yields a negligible decrease in effective dose compared to riskTCM. For iodine‐enhanced circular scans, all studied anatomical regions from shoulder to pelvis benefit from the additional tube voltage modulation, in particular if X‐ray generators with voltages down to 50kV were available. For the currently clinically available voltage range of 70 kV to 150kV, effective dose reductions for eccentric anatomies in the shoulder and the pelvis are considerable and are accompanied by a reduction of tube power demands compared to mAsTCM at 70 kV.

## CONFLICTS OF INTEREST STATEMENT

The authors declare no conflicts of interest.
